# Hypoxemia in patients with idiopathic or heritable pulmonary arterial hypertension

**DOI:** 10.1371/journal.pone.0191869

**Published:** 2018-01-29

**Authors:** Ghaleb Khirfan, Tawfeq Naal, Batool Abuhalimeh, Jennie Newman, Gustavo A. Heresi, Raed A. Dweik, Adriano R. Tonelli

**Affiliations:** 1 Department of Internal Medicine, Medicine Institute, Cleveland Clinic, Cleveland, OH, United States of America; 2 Pathobiology Department, Cleveland Clinic, Cleveland, OH, United States of America; 3 Department of Pulmonary and Critical Care Medicine, Respiratory Institute, Cleveland Clinic, Cleveland, OH, United States of America; Augusta University, UNITED STATES

## Abstract

**Background:**

The prevalence and prognostic implications of hypoxemia either at rest or during six-minute walk test (6MWT) in patients with idiopathic or heritable pulmonary arterial hypertension (IPAH or HPAH) have not been systemically studied.

**Objectives:**

We sought to determine the prevalence, phenotypic and prognostic implications of hypoxemia in patients with IPAH and HPAH.

**Methods:**

Patients with IPAH or HPAH were identified from the Cleveland Clinic Pulmonary Hypertension Registry. Pulse oximetry (SpO_2_) at rest and during 6MWT was used to define hypoxemia at rest or during activities when measurements were lower than 90%, respectively.

**Results:**

A total of 292 patients (age 50.6 ± 18.0 years, 73% females) with IPAH (88%) and HPAH (12%) were included. Of them, 143 (49%) had SpO_2_ >90% at rest and during 6MWT, 89 (31%) subjects had hypoxemia during 6MWT and 60 (20%) had hypoxemia at rest. Patients with hypoxemia had older age, greater body mass index, higher prevalence of cardiovascular risk factors, worse functional capacity and pulmonary function tests but less severe pre-capillary pulmonary hypertension. Individuals with hypoxemia either at rest or during the initial 6MWT had worse long-term survival when compared to subjects without hypoxemia, even when adjusting for a great number of potential confounders. (HR: 2.5 (95% CI: 1.54–3.98))

**Conclusions:**

Hypoxemia in patients with IPAH and HPAH is associated with more comorbidities, less severe pre-capillary pulmonary hypertension and worse survival.

## Introduction

Idiopathic and heritable pulmonary artery hypertension (IPAH and HPAH) are conditions characterized by increased pulmonary vascular resistance which can lead to right heart failure and premature death [1]. Patients with IPAH and HPAH can present with hypoxemia at rest or during activities, given lower mixed venous oxygenation, abnormalities in the ventilation / perfusion relationship and right-to-left shunt in individuals with patent foramen ovale. In addition, patients with IPAH and HPAH can experience comorbidities or overlap with other pulmonary hypertension groups [2]; factors that could contribute to the development of hypoxemia.

Blood gas abnormalities, i.e. hypoxemia and hypocapnia, were described in patients with IPAH included in the National Institute of Health (NIH) Registry. The mean ± standard deviation (SD) arterial partial pressure of O_2_ (PaO_2_) was 70 ± 13 mmHg in men and 72 ± 16 mmHg in women [3]. In this registry, there was a suggestion that PaO_2_ could predict survival in univariable analysis (hazard ratio (HR) with 95% CI: 0.98 (0.97–1.00)) [4]. Hoeper et al. retrospectively reviewed arterialized capillary blood gases in patients with IPAH without patent foramen ovale (PFO). These authors reported a mean ± SD PaO_2_ and arterial oxygen saturation (SaO_2_) on room air of 69 ± 14 mmHg and 93 ± 4%, respectively; measurements that did not significantly impact survival [5].

Although mild hypoxemia appears to be common in IPAH [5], the true prevalence and phenotypic characteristics of patients with IPAH or HPAH with either hypoxemia at rest or during six-minute walk test (6MWT) have not been systematically investigated. In addition, the prognostic implications of hypoxemia at rest or during activities, particularly when adjusted by confounders, remains unclear in this cohort of patients. We hypothesized that hypoxemia at rest or during 6MWT is common in patients with IPAH or HPAH and is associated with more comorbidities with clinical implications.

## Methods

### a) Study subjects

This retrospective study was approved by the Cleveland Clinic institutional review board (study number 16–452). Written informed consent was waived. Patients were identified from the Cleveland Clinic Pulmonary Hypertension Registry. We retrospectively included consecutive patients newly diagnosed with either IPAH or HPAH [6] from January 2000 to July 2015. All patients underwent right heart catheterization (RHC) and had evidence of pulmonary arterial hypertension (PAH) characterized by a mean pulmonary artery pressure (mPAP) ≥ 25 mmHg, pulmonary artery wedge pressure (PAWP) ≤ 15 mmHg and pulmonary vascular resistance (PVR) > 3 Wood units [2]. Two pulmonary hypertension experts reviewed the information available and agreed on the PAH etiology based on current guidelines [6]. We excluded patients with more than mild emphysema or interstitial lung disease on radiographic studies and more than moderate degree of obstruction or restriction on pulmonary function tests. Patients with moderate obstruction or restriction on pulmonary function tests were included only if the pulmonary hypertension was not explained by the underlying parenchymal or chronic obstructive lung disease.

### b) Pulse oximetry

The 6MWT was routinely performed in our outpatient clinic during the first visit to any of our PH specialist. The 6MWT was performed following American Thoracic Society (ATS) standards [7, 8] with the addition of pulse oximetry. We used pulse oximetry for the rapid noninvasive assessment of oxygenation at the time of 6MWT. Patients remained in a sitting position for at least 10 minutes before the resting SpO_2_ was measured. During the 6MWT, we continuously measured the SpO_2_ and recorded the lowest value that was reproducible in the setting of an adequate waveform. Whenever possible we used a finger probe. Nail polish was routinely removed. A forehead probe was used in cases of poor circulation or unreliable reading. We strove to avoid any potential artifacts and paid particular attention to the quality of the SpO_2_ waveform. The validity of the SpO_2_ measurements was determined based on the waveform quality and correlation with the patient’s heart rate [9].

In the present study, hypoxemia at rest was considered present when the resting SpO_2_ on room air at the time of the initial 6MWT was ≤ 89% or the patient had previously qualified for continuous O_2_ supplementation by Medicare guidelines (resting SpO_2_ ≤ 88% or PaO_2_ ≤ 55 mmHg or SpO_2_ of 89% or PaO_2_ between 56 and 59 mmHg on room air and evidence of congestive heart failure, cor pulmonale or hematocrit > 56%). Hypoxemia during 6MWT was considered present when a reliable SpO_2_ measurement was ≤ 89% at any point during the walking portion of the test.

### c) Arterial blood gas analysis

Both paper and electronic charts were reviewed to obtain data on the arterial blood gases (ABG) performed the closest to the date of PAH diagnosis. Blood samples were obtained from the radial artery in the sitting position after the patient rested for at least 5 minutes. In every patient, we recorded the amount of O_2_ supplementation administered at the time of the ABG. ABG determinations were performed with the ABL800 FLEX Blood Gas Analyzer (Radiometer, Brønshøj, Denmark). In contrast to SpO_2_ during 6MWT, ABG determination were not part of the tests routinely ordered during the first outpatient visit for pulmonary hypertension.

### d) Clinical and laboratory data at the time of initial six-minute walk test

We collected data on demographics, smoking status, co-morbidities, New York Heart Association (NYHA) functional class, N-terminal pro B-type natriuretic peptide (NT-proBNP), pulmonary function tests (PFTs), imaging studies (CXR and CT chest), transthoracic echocardiography with agitated saline contrast, and diagnostic RHC at the time of the initial 6MWT. We carefully recorded whether patients were on PAH-specific therapies at the time of the initial 6MWT. Patients could be receiving PAH-specific therapies at the time of the initial 6MWT if they could not walk immediately before the initiation of treatment or patients had started on PAH-specific therapies at outside practices or institutions.

### Statistical analysis

Continuous variables are summarized using mean ± standard deviation (SD) or median and interquartile range (IQR) when appropriate. Numerical variables were compared using t-test or Wilcoxon signed rank test. Categorical variables were compared with chi-square or Fischer’s exact test. Survival analysis was performed with Kaplan-Meier and groups were compared with log-rank test. In addition, we adjusted the survival analyses using the Cox proportional hazards model. The starting point for all the survival analyses was the date of the initial 6MWT. Patients were censored at the time of lung transplantation and followed until death or end of the study in January 2017. Cox proportional hazards model results are expressed as hazard ratios (HR) with the corresponding 95% confidence intervals (CI). All p values were reported as two tailed and a value of <0.05 was considered statistically significant. Statistical analyses were performed using the statistical packages SPSS version 17 (IBM; Armonk, N.Y., USA) or MedCalc version 14.12.2 (MedCalc Software bvba, Ostend, Belgium).

## Results

### Patient characteristics

**Fig 1:**
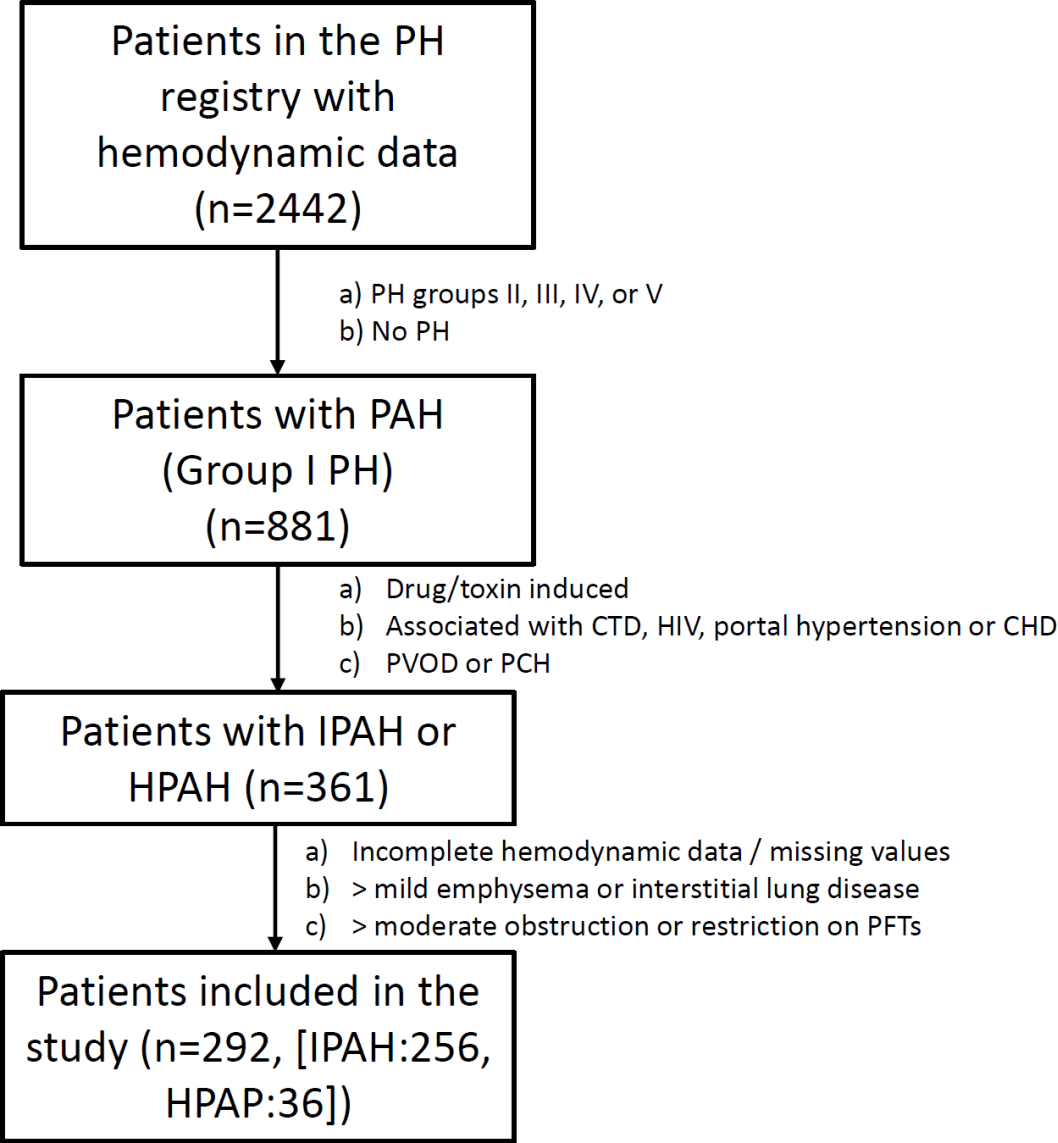
Selection of study participants. **Definition of abbreviations**: CHD: congenital heart disease, CTD: connective tissue disease, HIV: human immunodeficiency virus, HPAH: heritable pulmonary arterial hypertension, IPAH: idiopathic pulmonary arterial hypertension, PAH: pulmonary arterial hypertension, PCH: pulmonary capillary hemangiomatosis, PFTs: pulmonary function tests, PH: pulmonary hypertension, PVOD: pulmonary veno-occlusive disease.

We included a total of 292 patients (Fig 1) with a mean ± SD age of 50.6 ± 18.0 years, of whom 214 (73%) were women. The etiology of PAH was idiopathic in 256 patients (88%) and heritable in 36 patients (12%). The rest of the patients’ baseline characteristics are shown in Table 1. There was a subset of patients (n = 71, 24%) who were receiving PAH-specific therapies at the time of the index 6MWT and these therapies were initiated a median (IQR) of 3.5 (1 to 14) months before this test.

**Table 1:** Patient characteristics at the time of the initial 6MWT based on SpO2 at rest and during the 6MWT.

Variable	SpO2 ≥ 90% at rest and during 6MWT (n = 143)	SpO2 < 90% during 6MWT (n = 89)	SpO2 < 90% at rest (n = 60)	P (ANOVA, Chi square)
Age, yr	44.8 ± 16.9	53.2 ± 19.6	60.7 ± 11.5	<0.001
Female gender, n (%)	112 (78)	57 (64)	45 (75)	0.05
White race, n (%)	115 (51)	63 (77)	48 (86)	0.39
BMI(kg/m^2^)	29.7 ± 7.9	29.0 ± 8.2	32.5 ± 7.3	0.03
Syncope, n (%)	38 / 131 (29)	13 / 65 (20)	5 / 46 (11)	0.03
Raynaud phenomenon, n (%)	3 / 133 (2)	4 / 66 (6)	1 / 46 (2)	0.32
DM type 2, n (%)	16 / 131 (12)	21 / 76 (28)	18 / 52 (35)	0.001
HTN, n (%)	51 / 132 (39)	42 / 76 (55)	37 / 52 (71)	<0.001
Hypercholesterolemia, n (%)	18 / 119 (15)	25 / 57 (44)	21 / 40 (53)	<0.001
OSA, n (%)	25 / 119 (21)	15 / 54 (28)	15 / 40 (38)	0.11
CAD, n (%)	9 / 119 (8)	13 / 57 (23)	14 / 42 (33)	<0.001
Smoking history				
Current, n (%)	25 (18)	12 (15)	5 (9)	<0.001
Former, n (%)	37 (26)	28 (34)	36 (64)	
Never, n (%)	80 (56)	42 (51)	15 (27)	
Any PH medication, n (%)	26 (18)	23 (26)	21 (36)	0.03
NYHA class IV, n (%)	22 (17)	14 (19)	20 (38)	0.008
**6MWT**				
Resting HR (BPM)	83.7 ± 14.4	80.8 ± 14.0	82.2 ± 13.4	0.33
Maximum HR (BPM)	123.0 ± 21.0	115.3 ± 19.7	108.0 ± 19.6	<0.001
Distance walked (m)	337 ± 113	302 ± 113	212 ± 101	<0.001
Distance walked (% predicted)	60 ± 19	58 ± 19	44 ± 22	<0.001
**PFT**				
FVC (% predicted)	84 ± 16	81 ± 19	74 ± 20	0.003
FEV_1_ (% predicted)	80 ± 15	74 ± 19	67 ± 19	<0.001
FEV_1_/FVC	0.79 ± 0.10	0.75 ± 0.10	0.72 ± 0.10	<0.001
TLC (% predicted)	91 ±13	87 ± 14	84 ± 15	0.02
DLCO (% predicted)	71 ± 18	53 ± 22	38 ± 18	<0.001
**Echocardiogram**				
PFO (yes), n (%)	28 / 86 (33)	31 / 60 (52)	15 / 45 (33)	0.046
Intrapulmonary shunt (yes), n (%)*	5/58 (9)	1/29 (3)	5/30 (17)	0.21
RVSP (mmHg)	78 ± 22	84 ± 25	78 ± 28	0.19
**CXR**				
Increase interstitial markings, n (%)	9 / 134 (7)	10 / 84 (12)	10 / 55 (18)	0.001
Pleural effusion, n (%)	6 /134 (4)	3 / 84 (4)	5 / 55 (9)	0.31
**CT chest**				
GGOs, n (%)	31 / 104 (30)	17 / 62 (27)	12 / 46 (26)	0.87
Emphysema, n (%)	7 / 104 (7)	5 / 63 (8)	15 / 46 (33)	<0.001
Increase interstitial markings, n (%)	9 / 104 (9)	11 / 62 (17)	8 / 46 (13)	0.17
Pleural effusion, n (%)	5 / 104 (5)	5 / 63 (8)	2 / 46 (4)	0.64
**Laboratory**				
NT-pro BNP (pg/ml)	1115 ± 1929	3299 ± 6414	2376 ± 2409	0.048
**RHC**				
RA pressure (mmHg)	11.0 ± 6.6	10.3 ± 6.0	11.4 ± 6.4	0.55
Mean PAP (mmHg)	54.9 ± 12.9	52.3 ± 14.1	51.3 ± 10.5	0.12
PAWP (mmHg)	9.7 ± 3.8	9.8 ± 3.7	10.3 ± 3.7	0.58
TPG (mmHg)	45.1 ± 12.6	42.2 ± 13.8	40.6 ± 10.5	0.047
DPG (mmHg)	28.1 ± 11.6	25.4 ± 12.2	23.4 ± 9.6	0.02
CI (L/min/m2) by thermodilution	2.2 ± 0.7	2.2 ± 0.6	2.3 ± 0.6	0.23
PVR (Wood units)	12.7 ± 7.1	12.2 ± 6.9	10.0 ± 4.4	0.03
SvO_2_ (%)	61.5 ± 9.3	62.2 ± 9.0	61.9 ± 7.7	0.89

**Definition of Abbreviations:** BMI: body mass index, BP: blood pressure, BPM: beats per minute, CAD: coronary artery disease, CI: cardiac index, COPD: chronic obstructive pulmonary disease, DLCO: diffusion lung capacity for carbon monoxide, DM: diabetes mellitus, DPG: diastolic pulmonary gradient, FEV1: forced expiratory volume in 1 second, FVC: forced vital capacity, GGOs: ground glass opacities, HR: heart rate, HTN: hypertension, ILD: interstitial lung disease, NT-pro BNP: N-terminal pro B-type natriuretic peptide, NYHA: New York Heart Association functional class, OSA: obstructive sleep apnea, PAP: pulmonary artery pressure, PAWP: pulmonary artery wedge pressure, PFO: patent foramen ovale, PFT: pulmonary function test, PH: pulmonary hypertension, PVR: pulmonary vascular resistance, RA: right atrial, RHC: right heart catheterization, RVSP: right ventricular systolic pressure, SpO2: pulse oximeter oxygen saturation, SvO2: mixed venous oxygen saturation, TLC: total lung capacity, TPG: transpulmonary pressure gradient, 6MWT: six-minute walk test

Median time and interquartile range (IQR) in months between index 6MWT and the following tests were: PFT: 0 (-1 to 0) months, CXR: 0 (0 to 0) months), CT chest: 0 (-1 to 3) months and RHC: 0 (-3 to 0) months. Data expressed as mean ± SD unless otherwise indicated. * Intrapulmonary shunt was considered present when microbubbles were seen in the left atrium after the third cardiac cycle from their first appearance in the right atrium.

### Pulse oximetry

A total of 143 (49%) patients had a SpO_2_ ≥ 90% both at rest and during the initial 6MWT, meanwhile, 89 (31%) patients had hypoxemia only during the 6MWT and 60 (20%) patients had hypoxemia at rest. Of the 60 patients with hypoxemia at rest, 16 were on continuous O_2_ supplementation (O_2_ flow of 4.9 ± 1.5 L/min, range: 2–6 L/min). In the 44 patients with resting hypoxemia not on O_2_ supplementation, the mean SpO_2_ was 84.8 ± 4.5% (range 66–89%). Patients with hypoxemia during the initial 6MWT had a SpO_2_ during the activity of 86.4 ± 4.0% (range 74 to 89%). In the subgroup of patients not on any PAH-specific therapies at the time of the initial 6MWT, 117 (53%), 66 (30%) and 38 (17%) individuals had SpO_2_ ≥ 90% both at rest and during 6MWT, hypoxemia during the 6MWT or hypoxemia at rest, respectively (S1 Table in the online data supplement).

Arterial blood gases were available in 191 (65%) patients. A total of 124 (42%) patients had ABG obtained while breathing room air. The median (IQR) time between the ABG and the initial 6MWT was 0 (-8 to +1) months. ABG results are shown in Table 2.

**Table 2:** Arterial blood gases obtained on room air based on SpO_2_ at rest and during the initial 6MWT.

Variable	All study subjects (n = 124)	SpO2 ≥ 90% at rest and during 6MWT (n = 61)	SpO2 < 90% during 6MWT (n = 39)	SpO2 < 90% at rest (n = 24)	P (ANOVA)
pH	7.44 ± 0.06	7.45 ± 0.04	7.43 ± 0.08	7.46 ± 0.04	0.19
PaCO_2_ (mmHg)	34.0 ± 5.8	33.5 ± 6.1	34.1 ± 5.9	35.1 ± 4.8	0.49
PaO_2_ (mmHg)	65.0 ± 16.2	71.5 ± 15.4	61.6 ± 14.6	54.2 ± 13.5	< 0.001
SaO_2_ (%)	87.7 ± 9.5	90.9 ± 6.9	85.6 ± 12.8	83.8 ± 6.4	0.003
COHb (%)	1.5 ± 1.1	1.37 ± 1.18	1.60 ± 1.11	1.46 ±1.13	0.69
MetHb (%)	0.72 ± 0.31	0.73 ± 0.30	0.67 ± 0.35	0.75 ± 0.29	0.66
Hb (g/dl)	13.9 ± 2.4	14.1 ± 2.4	14.1 ± 2.6	13.3 ± 2.0	0.53

**Definition of Abbreviations:** COHB: carboxyhemoglobin, Hb: hemoglobin, MetHb: methemoglobin, PaCO2: partial pressure of carbon dioxide, PaO2: partial pressure of oxygen, SpO2: pulse oximeter oxygen saturation, SaO2: arterial oxygen saturation, 6MWT: six-minute walk test.

Data expressed as mean ± SD unless otherwise indicated.

### Group comparison

Patients with SpO_**2**_ < 90% at rest were significantly older, had higher body mass index(BMI) and prevalence of diabetes mellitus type 2, systemic hypertension, hypercholesterolemia, coronary artery disease than individuals with SpO_**2**_ ≥ 90%. Subjects with resting hypoxemia had worse NYHA functional class, walked less during 6MWT and had lower forced expiratory volume in 1 second (FEV_**1**_), total lung capacity and diffusion lung capacity for carbon monoxide. Imaging studies in patients with hypoxemia showed more pronounced interstitial markings and emphysema; meanwhile, these patients appeared to have less severe pulmonary hemodynamics supported by a lower pulmonary vascular resistance (PVR), transpulmonary and diastolic pressure gradients. Interestingly, most of the differences occurred in a graded fashion from patients without hypoxemia, hypoxemia during 6MWT and hypoxemia at rest (Table 1). Patients with hypoxemia compared to those without, more commonly received PAH-specific treatments at the time of the initial 6MWT (29.5 vs 18%, p = 0.03). A sensitivity analysis showed that the previously mentioned differences persisted across the three groups of patients, when only including treatment naïve subjects at the time of their initial 6MWT (S1 Table in the online data supplement).

### Correlation between presence of hypoxemia and survival

**Fig 2:**
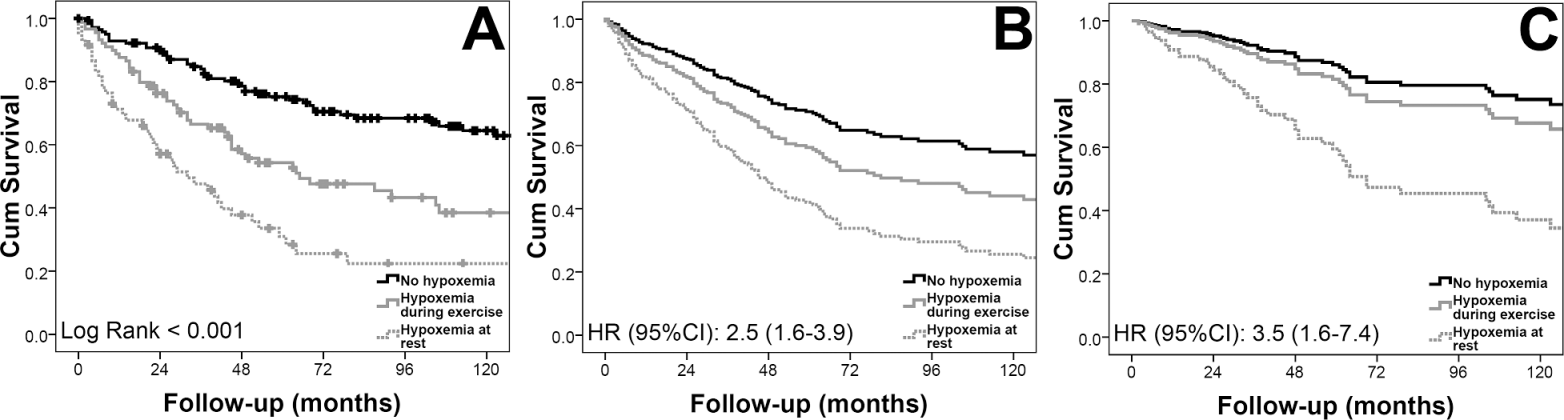
Kaplan-Meier and Cox survival analysis for the entire cohort.

The overall median survival was 105 (95% CI: 61–149) months, with 86%, 79% and 59% of the patients alive at 1, 2 and 5 years, respectively. For patients with SpO_2_ ≥ 90% at rest and during 6MWT, the median survival was 207 (95% CI: 153–261) months. The survival decreased to 65 (26–104) and 32 (17–47) months for those with hypoxemia during initial 6MWT or at rest, respectively (log rank test: <0.001) (Fig 2, panel A, see Table 3 for number of patients at risk). The presence of hypoxemia either during the initial 6MWT or at rest was associated with worse survival in a Cox survival analysis adjusted by age and gender (HR: 2.8 (95% CI: 1.82–4.33)). This increased mortality risk persisted after including in the model PVR and treatment with PAH-specific therapies (HR: 2.50, CI: 1.59–3.92, p<0.001) (Fig 2, panel B), adding BMI and smoking status (HR: 2.57, 95% CI: 1.55–4.25, p<0.001), and incorporating FEV_1_ and previous diagnosis of diabetes mellitus, systemic hypertension, hypercholesterolemia, sleep apnea and coronary artery disease (HR: 3.47, 95% CI: 1.63–7.35, p = 0.001) (Fig 2, panel C).

**Table 3:** Fig 2 Panel A foot table: Number of patients at risk.

Time (months)	0	24	48	72	96	120
No hypoxemia, n	143	123	96	72	61	42
Hypoxemia during 6MWT, n	89	64	41	26	19	14
Hypoxemia at rest, n	60	32	18	9	6	5

When survival was adjusted by age, gender, PVR and use of PAH-specific medications (panel B), there was an increase in mortality in patients with hypoxemia during the initial 6MWT compared to patients without hypoxemia (HR: 1.56, CI: 1.01–2.41, p = 0.04). Similarly, there was a higher risk of dying in individuals with hypoxemia at rest than during the six-minute walk (HR: 1.58, CI: 1.00–2.51, p = 0.049).

**Fig 3:**
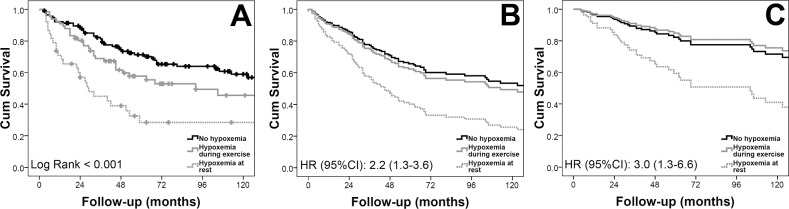
Kaplan-Meier and Cox survival analysis for cases not on PAH-specific therapies.

In a sensitivity analysis that included treatment naïve patients (n = 221), we noted a worse survival for those individuals with hypoxemia (log rank test: <0.001) (Fig 3, panel A, see Table 4 for number of patients at risk). The difference in survival persisted in a Cox survival analysis adjusted for age, gender and PVR (HR for the presence of hypoxemia during 6MWT or at rest: 2.17, 95% CI: 1.30–3.64, p = 0.003) (Fig 3, panel B), even with the addition of BMI, smoking status, FEV_1_, and prior diagnosis of diabetes mellitus, systemic hypertension, hypercholesterolemia, sleep apnea and coronary artery disease (HR: 2.96, 95% CI: 1.31–6.62, p = 0.009) (Fig 3, panel C).

**Table 4:** Fig 3 Panel A foot table: Number of patients at risk.

Time (months)	0	24	48	72	96	120
No hypoxemia, n	117	99	72	52	45	30
Hypoxemia during 6MWT, n	66	51	32	19	13	11
Hypoxemia at rest, n	38	20	12	6	5	4

### Sensitivity analysis including patients with no more than mild PFTs abnormalities

When including patients with no more than mild PFTs abnormalities (n = 197 of 279 patients with PFTs data available, 71%) we noted that 118 (60%) had SpO_2_ ≥ 90% at rest and during 6MWT, 53 (27%) had SpO_2_ < 90% during 6MWT and 26 (13%) had SpO_2_ < 90% at rest; representing a small decrease in the prevalence of hypoxemia during 6MWT and at rest (3.3% and 6.6%, respectively). The presence of hypoxemia during 6MWT or at rest continued to be associated with worse survival (log rank test: <0.001) even when adjusted by age, gender, PVR, treatment with PAH-specific therapies, BMI and previous diagnosis of diabetes mellitus, systemic hypertension, hypercholesterolemia, sleep apnea and coronary artery disease (HR: 3.06, 95% CI: 1.63–5.71, p<0.001)

## Discussion

In a cohort of consecutive patients with well characterized IPAH and HPAH, in whom three quarters were treatment naïve, we noted that at least half of the individuals had some degree of hypoxemia (SpO_2_ < 90%) either at rest or during the initial 6MWT. Patients with hypoxemia had a phenotype characterized by older age, greater BMI, higher prevalence of cardiovascular risk factors, worse functional capacity and pulmonary function tests but less severe pre-capillary pulmonary hypertension. Individuals with hypoxemia either at rest or during the initial 6MWT had worse long-term survival when compared to subjects without hypoxemia, even after adjusting for a great number of potential confounders.

In the present study, we used reliable pulse oximetry results both at rest and during 6MWT, to define the oxygenation status. Pulse oximetry estimates peripheral arterial O_2_ saturation and this measurement may differ by 2 to 4% points from the arterial O_2_ saturation obtained by ABG [10, 11]. Although pulse oximetry is not the gold standard to determine oxygenation, this methodology provides a simple and noninvasive assessment which is routinely used in medical practice [12]. Pulse oximetry also allows a continuous assessment of the oxygenation during 6MWT [8]. In contrast, ABG are not routinely obtained in patients with PAH given its invasive nature. In our practice, ABG are not consistently obtained at the time of diagnosis of IPAH, therefore the reasons for obtaining this test were diverse. Given this important limitation, we decided to focus our analyses on the results obtained by pulse oximetry and not ABG analysis.

It appears that patients with hypoxemia at rest and during 6MWT have a phenotype characterized by older age, higher BMI, greater prevalence of comorbidities and less severe pre-capillary pulmonary hypertension. Trip et al. showed that a group of IPAH patients with lower DLCO (<45% of predicted) had a lower arterial oxygen saturation at rest, greater decrease in oxygen saturation during 6MWT, were older with lower functional capacity and had greater prevalence tobacco exposure and comorbidities [13]. It is unclear whether some components of this phenotype are a cause or consequence of the hypoxemia. For instance, patients with hypoxemia may limit their physical activity which could lead to obesity and subsequent increase in the cardiovascular risk factors and other associated comorbidities. Alternatively, it is possible that patients with IPAH or HPAH who are diagnosed at an older age have a higher prevalence of comorbidities, some of them associated with hypoxemia such as obesity with atelectasis, and mild/moderate COPD or interstitial lung disease. These comorbidities which are relatively common in the general population, may contribute to the development of hypoxemia in patients with IPAH or HPAH. Although the patients included in our study had IPAH or HPAH, supported by two pulmonary hypertension specialists, it is certainly possible that some individuals had a variable degree of overlap with other pulmonary hypertension groups 2 or 3[6].

Hypoxemia during exercise has been associated with lower functional capacity and worse prognosis in patients with COPD [14, 15] or interstitial lung disease [16, 17]. In our cohort, hypoxemia either at rest or during the initial 6MWT tracked with worse survival even after adjusting for traditional confounders such age, gender, comorbidities, hemodynamic severity and treatment with PAH-specific therapies at the time of the initial 6MWT. Given that hypoxemia is mitigated with O_2_ supplementation, it is unlikely that this condition per se significantly affected survival to the degree observed in our study. It is more probable that the hypoxemia represents one of the characteristics of a phenotype linked to worse survival. Paciocco et al. showed that in patients with IPAH (n = 34), a lower SpO_2_ at peak 6MWT distance and a reduction in SpO_2_ during activity were associated with increased mortality [18]. Hypoxemia during 6MWT could be due to a reduced time for capillary gas exchange and/or opening of patent foramen ovale (PFO) with right-to-left shunt [19, 20]. In our study, the prevalence of PFO diagnosed with contrast-enhanced transthoracic echocardiograph appeared to be higher in individuals with hypoxemia during the 6-minute walk but not at rest. The reasons for this finding are unclear at this point.

Our study has limitations that include a) retrospective data collection, b) the diagnosis of hypoxemia was made using pulse oximetry, and c) a quarter of patients were receiving PAH-specific therapies at the time of the initial 6MWT. Although we included patients on PH therapies to be comprehensive, we also performed sensitivity analyses in treatment naïve incident cases. In spite of these limitations our study is the first to systematically explore the prevalence, phenotypic characteristics and long-term survival implications in well characterized patients with IPAH or HPAH and hypoxemia at rest or during initial 6MWT.

## Conclusions

Patients with IPAH or HPAH who have hypoxemia at rest or during 6MWT are older, with worse functional capacity, more pronounced cardiovascular and respiratory co-morbidities, and a less severe pre-capillary pulmonary arterial hemodynamics. Remarkably, this hypoxemic phenotype with higher number of comorbidities and possible overlap with other pulmonary hypertension groups conveys a higher mortality.

## Supporting information

S1 Table Patient characteristics based on SpO_2_ at rest and during 6MWT in treatment naïve subjects. (DOCX)
