# Supplementary material for: Hypoxemia in patients with idiopathic or heritable pulmonary arterial hypertension
Source: PLoS One. 2018 Jan 29;13(1):e0191869. doi: 10.1371/journal.pone.0191869 (PMC5788375; doi:10.1371/journal.pone.0191869)
Supplement: S1 Table — (DOCX) [file pone.0191869.s001.docx]

S1 Table: Patient characteristics based on SpO_2_ at rest and during 6MWT in treatment naïve subjects

| **Variable** | **SpO2 ≥ 90% at rest and during 6MWT**  (n= 117) | | | **SpO2 < 90% during 6MWT**  (n= 66) | | **SpO2 < 90% at rest**  (n= 38) | **P**  **(ANOVA, Chi square)** |
| --- | --- | --- | --- | --- | --- | --- | --- |
| Age, yr | 44.7 ± 17.7 | | 54.1 ± 19.2 | | 61.3 ± 10.2 | | <0.001 |
| Female gender, n (%) | 91 (78) | | 43 (65) | | 28 (74) | | 0.18 |
| White race, n (%) | 94 (81) | | 45 (76) | | 29(83) | | 0.22 |
| BMI(kg/m^2^) | 29.3 ± 7.7 | | 28.7 ± 8.4 | | 32.1 ± 6.8 | | 0.10 |
| Syncope, n (%) | 31 / 107 (29) | | 9 / 49 (18) | | 3 / 29 (10) | | 0.07 |
| DM type 2, n (%) | 13 / 105 (12) | | 15 / 54 (28) | | 9 / 31 (29) | | 0.02 |
| HTN, n (%) | 40 / 106 (38) | | 31 / 54 (57) | | 23 / 31 (74) | | <0.001 |
| Hypercholesterolemia, n (%) | 13 / 94 (14) | | 20 / 46 (43) | | 16 / 25 (64) | | <0.001 |
| OSA, n (%) | 17 / 94 (18) | | 12 / 44 (27) | | 8 / 25 (32) | | 0.23 |
| CAD, n (%) | 8 / 94 (8) | | 10 / 46 (23) | | 8 / 26 (33) | | 0.01 |
| Smoking history |  | |  | |  | |  |
| Current, n (%) | 23 (20) | | 9 (14) | | 4 (11) | | 0.001 |
| Former, n (%) | 27 (23) | | 21 (32) | | 23 (61) | |  |
| Never, n (%) | 66 (56) | | 33 (50) | | 9 (24) | |  |
| NYHA class IV, n (%) | 18 (18) | | 10 (17) | | 12 (34) | | 0.08 |
| **6MWT** | | | | | | | |
| Resting HR (BPM) | 83.0 ± 15.0 | | 80.3 ± 13.4 | | 82.4 ± 13.0 | | 0.48 |
| Maximum HR (BPM) | 121.9 ± 21.3 | | 116.6 ± 20.5 | | 106.9 ± 19.3 | | 0.001 |
| Distance walked (m) | 330 ± 112 | | 307 ± 115 | | 198 ± 108 | | <0.001 |
| Distance walked (% predicted) | 59 ± 18 | | 59 ± 20 | | 42 ± 22 | | <0.001 |
| **PFT** | | | | | | | |
| FVC (% predicted) | 84 ± 17 | | 82 ± 17 | | 75 ± 21 | | 0.06 |
| FEV_1_ (% predicted) | 80 ± 16 | | 77 ± 18 | | 69 ± 19 | | 0.002 |
| FEV_1_/FVC | 0.80 ± 0.10 | | 0.76 ± 0.10 | | 0.72 ± 0.10 | | <0.001 |
| TLC (% predicted) | 91 ±13 | | 87 ± 14 | | 87 ± 16 | | 0.24 |
| DLCO (% predicted) | 71 ± 19 | | 55 ± 23 | | 39 ± 20 | | <0.001 |
| **Echocardiogram** | | | | | | | |
| PFO (yes), n (%) | 23 / 71 (32) | | 28 / 51 (55) | | 11 / 29 (38) | | 0.04 |
| RVSP (mmHg) | 77 ± 21 | | 82 ± 22 | | 79 ± 27 | | 0.38 |
| **CXR** | | | | | | | |
| Increase interstitial markings, n (%) | 6 / 108 (6) | | 7 / 61 (11) | | 7 / 35 (20) | | 0.04 |
| Pleural effusion, n (%) | 5 /108 (4) | | 2 / 61 (3) | | 3 / 35 (9) | | 0.50 |
| **CT chest** | | | | | | | |
| GGOs, n (%) | | 21 / 82 (26) | 13 / 51 (25) | | 9 / 31 (29) | | 0.92 |
| Emphysema, n (%) | | 6 / 82 (7) | 4 / 51 (8) | | 11 / 31 (35) | | <0.001 |
| Increase interstitial markings, n (%) | | 9 / 82 (11) | 8 / 51 (16) | | 4 / 31 (13) | | 0.73 |
| Pleural effusion, n (%) | | 3 / 82 (4) | 5 / 51 (10) | | 1 / 31 (3) | | 0.26 |
| **Laboratory** | | | | | | | |
| NT-pro BNP (pg/ml) | | 1190 ± 2032 | 3435 ± 6827 | | 1982 ± 2514 | | 0.09 |
| **RHC** | | | | | | | |
| RA pressure (mmHg) | | 11.0 ± 6.7 | 9.5 ± 5.6 | | 11.6 ± 6.1 | | 0.20 |
| Mean PAP (mmHg) | | 54.5 ± 12.8 | 51.2 ± 13.9 | | 51.5 ± 10.6 | | 0.12 |
| PAWP (mmHg) | | 9.7 ± 3.7 | 9.9 ± 4.0 | | 10.1 ± 3.8 | | 0.18 |
| TPG (mmHg) | | 44.5 ± 12.6 | 41.2 ± 13.6 | | 41.4 ± 11.2 | | 0.18 |
| DPG (mmHg) | | 27.7 ± 11.8 | 24.1 ± 12.1 | | 23.8 ± 9.5 | | 0.06 |
| CI (L/min/m2) thermodilution | | 2.2 ± 0.7 | 2.2 ± 0.6 | | 2.4 ± 0.7 | | 0.22 |
| PVR (Wood units) | | 12.6 ± 7.3 | 11.9 ± 6.5 | | 10.2 ± 4.9 | | 0.18 |
| SvO2 (%) | | 61.6 ± 9.6 | 63.3 ± 8.4 | | 62.1 ± 7.9 | | 0.55 |

**Definition of Abbreviations:** BMI: body mass index, BP: blood pressure, BPM: beats per minute, CAD: coronary artery disease, CI: cardiac index, COPD: chronic obstructive pulmonary disease, DLCO: diffusion lung capacity for carbon monoxide, DM: diabetes mellitus, DPG: diastolic pulmonary gradient, FEV1: forced expiratory volume in 1 second, FVC: forced vital capacity, GGOs: ground glass opacities, HR: heart rate, HTN: hypertension, ILD: interstitial lung disease, NT-pro BNP: N-terminal pro B-type natriuretic peptide, NYHA: New York Heart Association functional class, OSA: obstructive sleep apnea, PAP: pulmonary artery pressure, PAWP: pulmonary artery wedge pressure, PFO: patent foramen ovale, PFT: pulmonary function test, PH: pulmonary hypertension, PVR: pulmonary vascular resistance, RA: right atrial, RHC: right heart catheterization, RVSP: right ventricular systolic pressure, SpO2: pulse oximeter oxygen saturation, SvO2: mixed venous oxygen saturation, TLC: total lung capacity, TPG: transpulmonary pressure gradient, 6MWT: six-minute walk test

Data expressed as mean ± SD unless otherwise indicated.
